# Validation of the Dutch language version of the Safety Attitudes Questionnaire (SAQ-NL)

**DOI:** 10.1186/s12913-016-1648-3

**Published:** 2016-08-15

**Authors:** Marck HTM Haerkens, Wouter van Leeuwen, J. Bryan Sexton, Peter Pickkers, Johannes G. van der Hoeven

**Affiliations:** 1 Department of Intensive Care Medicine, Radboud University Medical Center, PO Box 9101, 6500 HB Nijmegen, The Netherlands; 2 Wings of Care, Koepelweg 12, 5263 AS Vught, The Netherlands; 3 Faculty of Psychology University of Amsterdam, PO Box 15804, 1001 NH Amsterdam, The Netherlands; 4 Royal Netherlands Air Force, PO Box 8762, 4820 BB Breda, The Netherlands; 5 Department of Psychiatry, Duke University School of Medicine, Duke University Health System, Durham, NC USA; 6 Duke Patient Safety Center, Duke University Health System, Durham, NC USA

**Keywords:** Human factors, Crew Resource Management (CRM), Safety Attitudes Questionnaire (SAQ), Dutch hospital setting

## Abstract

**Background:**

As the first objective of caring for patients is to do no harm, patient safety is a priority in delivering clinical care. An essential component of safe care in a clinical department is its safety climate. Safety climate correlates with safety-specific behaviour, injury rates, and accidents. Safety climate in healthcare can be assessed by the Safety Attitudes Questionnaire (SAQ), which provides insight by scoring six dimensions: Teamwork Climate, Job Satisfaction, Safety Climate, Stress Recognition, Working Conditions and Perceptions of Management.

The objective of this study was to assess the psychometric properties of the Dutch language version of the SAQ in a variety of clinical departments in Dutch hospitals.

**Methods:**

The Dutch version (SAQ-NL) of the SAQ was back translated, and analyzed for semantic characteristics and content. From October 2010 to November 2015 SAQ-NL surveys were carried out in 17 departments in two university and seven large non-university teaching hospitals in the Netherlands, prior to a Crew Resource Management human factors intervention. Statistical analyses were used to examine response patterns, mean scores, correlations, internal consistency reliability and model fit. Cronbach’s α’s and inter-item correlations were calculated to examine internal consistency reliability.

**Results:**

One thousand three hundred fourteen completed questionnaires were returned from 2113 administered to health care workers, resulting in a response rate of 62 %. Confirmatory Factor Analysis revealed the 6-factor structure fit the data adequately. Response patterns were similar for professional positions, departments, physicians and nurses, and university and non-university teaching hospitals. The SAQ-NL showed strong internal consistency (α = .87). Exploratory analysis revealed differences in scores on the SAQ dimensions when comparing different professional positions, when comparing physicians to nurses and when comparing university to non-university hospitals.

**Conclusions:**

The SAQ-NL demonstrated good psychometric properties and is therefore a useful instrument to measure patient safety climate in Dutch clinical work settings. As removal of one item resulted in an increased reliability of the Working Conditions dimension, revision or deletion of this item should be considered. The results from this study provide researchers and practitioners with insight into safety climate in a variety of departments and functional positions in Dutch hospitals.

**Electronic supplementary material:**

The online version of this article (doi:10.1186/s12913-016-1648-3) contains supplementary material, which is available to authorized users.

## Background

To err is human. As a result, everything that a human being devises, uses, or does is prone to error and failure. As this challenges the “First: do no harm” principle of healthcare [1], it is imperative to assess the factors that impact patient safety.

Patient safety is regarded by the National Patient Safety Foundation as the avoidance, prevention, and amelioration of adverse events or injuries stemming from the processes of healthcare [2]. Identifying the key factors in safe clinical care is a challenging task.

Evidence from non-clinical [3] and clinical [4–8] critical environments suggests a positive relationship between safety culture, safety climate, and safety outcome. Safety culture is defined by the British Health & Safety Commission as “the product of individual and group values, attitudes, perceptions, competencies, and patterns of behavior that determine the commitment to, and the style and proficiency of, an organization’s safety management [9]. From an anthropological standpoint, “safety culture” is only measurable by careful, long-term observations. Therefore, in daily clinical practice, it may be more appropriate to use the term “safety climate”, which generally refers to the measurable components of safety culture such as management behaviors, safety systems, and employee perceptions of safety.

Safety climate can be determined by the Safety Attitudes Questionnaire (SAQ), a validated healthcare derivative of the Cockpit Management Attitudes Questionnaire [10] that has been adapted to various clinical settings [4, 11]. The initial extended version consists of 60 items including 30 core items that are identical in all clinical settings. The short form version includes only the 30 core items.

Previous factor analysis identified factors covering six domains of the safety climate: *Teamwork Climate* (six items) is the perceived quality of collaboration between personnel. *Job Satisfaction* (five items) is defined as positivity about the work experience. *Safety Climate* (seven items) is the perception of a strong and proactive organizational commitment to safety. *Stress Recognition* (four items) is acknowledgement of how performance is influenced by stressors. *Working Conditions* (three items) is the perceived quality of the work environment and logistical support (such as staffing and equipment). *Perceptions Of Management* (five items) is the approval of managerial action [10]. SAQ responses are given on a 5-point Likert scale (1 = disagree strongly, 2 = disagree slightly, 3 = neutral, 4 = agree slightly, 5 = agree strongly). Two items (items 2,11) are reversed scored (https://med.uth.edu/chqs/surveys/safety-attitudes-and-safety-climate-questionnaire/).

Although the SAQ has been utilized in safety research in the Dutch care setting [6, 12, 13], no open source Dutch language version of the SAQ has been published to date. One exception is the observational study on the content validity and internal consistency of a Dutch translation of the SAQ by Devriendt and colleagues which was published during the course of our study [14]. Although good content validity (CVI = .83) and internal consistency (α = .90) were reported, the sample in the study was limited to, and conducted in, a single hospital in the culturally different context of Belgium [14]. Furthermore, even though Belgium and the Netherlands are neighboring countries the Dutch language differs from the Belgian-Dutch language (Flemish), which is clearly visible in the Belgian-Dutch questions. Contrary to our study, no certified interpreters and/or native English speakers performed the translation and the adapted Brislin protocol of forward and back translation was not used.

The Dutch hospital system consists of three levels of hospitals: large university hospitals, medium size non-university training hospitals and smaller rural hospitals.

The aim of the current study was to assess the psychometric properties of the Dutch language version of the SAQ (SAQ-NL) and provide insight into safety climate in a variety of departments and functional positions in Dutch hospitals.

## Methods

### Design and setting

From October 1^st^ 2010 to November 1^st^ 2015 a cross-sectional survey was conducted in 17 departments in two university and seven non-university teaching hospitals in the Netherlands as part of an intervention study evaluating the impact of Crew Resource Management (CRM) – human factors awareness training. This study focuses on the baseline data gathered before the CRM-training.

The clinical departments (and number of health care providers) that participated in this study included: two Intensive Care Units (ICU, *n* = 281), five Operating Rooms (OR, *n* = 648), two Cardiac Catheterization Labs (CCL, *n* = 56), one Medium Care Unit (MCU, *n* = 33), three Emergency Rooms (ER, *n* = 163), one Coronary Care Unit - Heart First Aid unit (CCU-HFA, *n* = 45), one Radiotherapy department (RTX, *n* = 12), one Department of Gastroenterology and Hepatology (DGH, *n* = 40) and one Pharmacy department (*n* = 36).

### The Safety Attitudes Questionnaire - NL

It was decided to use the original 30-item version of the SAQ benchmarked by Sexton et al. [10, 15] containing identical questions for all clinical settings as the basis for the Dutch version because of its usability in multiple clinical environments, good psychometric properties and open source accessibility.

When introducing a foreign language questionnaire, potential semantic and cultural differences need to be taken into account. To determine semantic equivalence (the translated items have the same meaning as in the original) in the translated version the SAQ was translated from English to Dutch and back again by native speakers (of which one is a certified interpreter) following the adapted Brislin protocol [16, 17]. The translated version was reviewed for semantic properties and content. A subject matter experts group, consisting of clinical faculty (*n* = 3), psychologists (*n* = 2) and human factors specialists (*n* = 3), analyzed clarity and appropriateness of wording and each item’s meaning in the cultural setting of the Netherlands.

### Data collection

All professionals of each participating department received an invitation to fill out the SAQ-NL. The first five departments were issued a paper and pencil version, all participants in subsequent departments received a link to an online questionnaire. There was no significant difference between the groups associated with method of administration.

### Statistical analysis

Frequency tables were generated to provide an overview of age categories, gender, professional positions, departments, department tenure, and hospital tenure of the responders. To provide an overview of response patterns, percentages for missing values (MV) were generated. Further analysis of MV was done by first recoding all MV to ‘0’ and all responses to ‘1’. These recoded values were then aggregated to yield an overall response score.

A univariate analysis of variance (ANOVA) was performed with the overall response score as dependent variable and profession and department as independent variables to check for differences in the overall response score. Independent *t*-tests were applied to compare the overall response scores between university and non-university hospitals and between medical staff (attending physicians and residents) and support personnel (nurses, operating room assistants, and operating room assistants). Mean scores were calculated per item and then aggregated to yield a mean score per SAQ dimension. Furthermore, to provide an overview of percentages of participants that agreed or disagreed with an item, responses of 1 and 2 on the 5-point scale were recoded as ‘disagree’ and responses 4 and 5 were recoded as ‘agree’.

Scale reliability analyses with all items and for each dimension separately resulted in a corrected item-total correlation and a Cronbach’s α if an item is deleted for the dimension-scale. An overview of missing values, means and standard deviations, percentages agree and disagree, corrected item-total correlations, Cronbach’s α’s, and Cronbach’s α’s if an item is deleted were calculated.

Based on the results of the factor analysis as performed earlier [10], a confirmatory factor analysis (CFA) was performed on participants who fully completed the instrument (*n* = 604).

CFA was performed with analysis of moment structures (AMOS) software [18].

We deemed a successful model was that with a Goodness of Fit Index (GFI) >0.9 [19], a Comparative Fit Index close to 0.95 [20] and a Root Mean Square Error of Approximation (RMSEA) <0.08 [21]. The χ^2^ statistic is also given (a poor measure of model fit of measurement, but included here for reasons of convention).

The unrestricted model was based on the structure of the original database. We fit a six factor unrestricted CFA model that contained the 30 items retained in the previous study of Sexton et al. [10] that confirmed the SAQ’s construct validity.

Mean scores and standard deviations for each SAQ-NL dimension were calculated for professional positions, physicians (residents and attending physicians) vs. nurses, departments, and academic status separately. Note that the category ‘nurses’ consists of nurses, operating room technicians, and anaesthesiology technicians. To explore whether groups differed on mean scores, multivariate analysis of variance (MANOVA) was utilized to interpret the mean scores. Because SPSS removes all participants with missing values in any combination of more than one independent variable, three separate MANOVA’s were performed with professional position, physicians vs. nurses, and university status of the hospital as independent variables and the mean scores on each dimension as dependent variables. Because dependent variables were not highly correlated and because it is robust to many violations of MANOVA, Pillai’s trace was utilized as the MANOVA test statistic [22].

Since no a priori hypotheses were formulated, a post-hoc Bonferroni test was utilized to interpret significant findings when the independent variable consisted of more than two groups. Finally, a bivariate correlation analysis was done to provide an overview of relations between SAQ-NL dimensions. For the correlation analysis, Pearson’s correlation was used with a two-tailed test of significance.

Because of the large statistical power due to large sample size, corresponding effect sizes are reported to interpret significant findings. The following cut-offs were used: small effect (Cohen’s *d* = 0.2, η_p_^2^ = 0.01), medium effect (*d* = 0.5, η_p_^2^ = 0.06), large effect (*d* = 0.8, η_p_^2^ = 0.14).

Data was analysed using SPSS Statistics 22 (IBM Corp., Armonk, NY, USA). A *p*-value <0.05 was considered to indicate significance.

## Results

### Demographics

One thousand three hundred fourteen of 2113 surveys were returned for a response rate of 62 %. This final sample consisted of 623 nurses (47 %), 239 attending physicians (18 %), 90 residents (6.8 %) and 214 “category other”(16 %). A total of 148 participants (11 %) did not provide their position details. The university hospitals (*n* = 2) employed 441 respondents, 873 respondents were employed by non-university teaching hospitals (*n* = 7). The database contained one outlier department with an exceptionally low response rate of 21 %.

Detailed demographic and professional characteristics of the responders are shown in Table 1.

**Table 1:** Frequency Table for Participant Demographic Variables, Departments, and Tenure

Age^a^		Gender		Position		Department		Tenure at department^a^		Tenure at hospital^a^	
Cat.	Freq. (%)	Cat.	Freq. (%)	Cat.	Freq. (%)		Freq. (%)	Cat.	Freq (%)	Cat.	Freq. (%)
≤20	25 (1.9)	Male	400 (30.4)	Nurse^b^	623 (47.4)	Intensive care	281 (21.4)	<1	122 (9.3)	<1	86 (6.5)
21–30	210 (16.0)	Female	813 (61.9)	Resident	90 (6.8)	Operating room	648 (49.3)	1–5	382 (29.1)	1–5	304 (23.1)
31–40	322 (24.5)			Att. physician	239 (18.2)	CCL	56 (4.3)	6–10	243 (18.5)	6–10	237 (18.0)
41–50	365 (27.8)			Other	214 (16.3)	Medium care	33 (2.5)	>10	476 (36.2)	>10	596 (45.4)
>50	304 (23.1)					Emergency room	163 (12.4)				
						CCU - HFA	45 (3.4)				
						Radiotherapy	12 (0.9)				
						DGH	40 (3.0)				
						Pharmacy	36 (2.7)				
Missing	88 (6.7)		101 (7.7)		148 (11.3)		0		91 (6.9)		91 (6.9)

*Note. N* = 1314; *Cat.* category, *Freq.* frequency, *Att. physician* attending physician, *CCL* cardiac catheterization lab, *CCU-HFA* coronary care unit - heart first aid unit, *DGH* Department of Gastroenterology and Hepatology; ^a^ Age and tenure in years; ^b^ Nurse category consists of nurses, operating room technicians, and anaesthesiology technicians

### SAQ-NL factor structure and multi-level modeling

The SAQ-NL with six factors and 30 items was used in all the administrations reported here. The 6-factor model fit the data well: χ^2^(390) = 931.18, *p* <0.001, GFI = 0.90, CFI = 0.91, and RMSEA = 0.05. Item loadings on respective factors appear in Additional file 1.

### SAQ-NL item characteristics

The subject matter experts adjusted the items until they agreed on the appropriateness of the semantic characteristics and deemed the content sufficient and appropriate for measuring safety climate in hospitals. Missing values (MV) analysis revealed a range of 3.0–6.8 % MV for the separate questions, see Additional file 1. ANOVA revealed no difference in MV for professional position, *F*(3, 1110) = 0.02, *p* = .996, or department, *F*(7, 1110) = 1.23, *p* = 0.283. Independent *t*-tests revealed no difference in MV for university status, *t*(1283.83) = 1.83, *p* = 0.059, and physicians vs. nurses *t*(950) = 0.75, *p* = 0.452. Due to a technical error, item 16 (“this is a good place to work”) did not appear in the questionnaire initially and therefore resulted in a MV of 50 %.

### SAQ-NL mean scores

An overview of mean scores and standard deviations for comparison is provided in Table 2. Using Pillai’s trace, the overall MANOVA’s revealed a medium effect of clinical position (*n* = 1159), *V* = 0.19, *F*(18, 3456) = 13.25, *p* <0.001, η_p_^2^ = 0.07, a large effect of physicians vs. nurses (*n* = 947), *V* = 0.14, *F*(6, 940) = 26.37, *p* <0.001, η_p_^2^ = 0.14, and a small effect of academic status of the hospital (*n* = 1257), *V* = 0.03, *F*(6, 1250) = 6.65, *p* <0.001, η_p_^2^ = 0.03, on the six SAQ-NL dimensions; Teamwork Climate, Safety Climate, Job Satisfaction, Stress Recognition, Perceptions of Management, and Working Conditions.

**Table 2:** SAQ-NL Means and (Standard Deviations) per Professional Position and Department

	Teamwork Climate	Safety Climate	Job Satisfaction	Stress Recognition	Perc. of Management	Working Conditions
Position						
Nurse	3.49 (0.55)*	3.37 (0.52)*	3.50 (0.64)*	2.97 (0.70)	2.89 (0.60)	3.23 (0.61)*
Resident	3.79 (0.47)*	3.50 (0.50)*	3.77 (0.51)*	2.92 (0.72)	3.03 (0.53)	3.49 (0.53)*
Attending physician	3.96 (0.49)*	3.72 (0.58)*	3.87 (0.62)*	3.07 (0.74)	2.96 (0.62)	3.49 (0.63)*
Other	3.47 (0.64)	3.52 (0.64)	3.75 (0.70)	2.98 (0.82)	3.01 (0.62)	3.24 (0.71)
Physician vs Nurse						
Physicians	3.91 (0.49)*	3.66 (0.57)*	3.85 (0.59)*	3.03 (0.73)	2.98 (0.60)*	3.49 (0.60)*
Nurses	3.49 (0.55)*	3.37 (0.52)*	3.50 (0.64)*	2.97 (0.70)	2.89 (0.60)*	3.23 (0.61)*
Department						
Intensive care	3.74 (0.47)	3.50 (0.44)	3.72 (0.49)	2.95 (0.60)	3.07 (0.51)	3.36 (0.52)
Operating room	3.48 (0.60)	3.41 (0.61)	3.48 (0.71)	3.00 (0.74)	2.84 (0.62)	3.28 (0.65)
CCL	3.76 (0.53)	3.71 (0.53)	4.01 (0.53)	2.79 (0.72)	3.39 (0.50)	3.07 (0.71)
Medium care	3.68 (0.37)	3.55 (0.37)	3.70 (0.33)	2.58 (0.48)	3.14 (0.44)	3.36 (0.43)
Emergency room	3.66 (0.54)	3.41 (0.52)	3.81 (0.59)	3.03 (0.74)	2.92 (0.61)	3.32 (0.57)
CCU - HFA	3.74 (0.63)	3.57 (0.74)	3.87 (0.58)	3.08 (0.93)	2.70 (0.64)	3.44 (0.77)
Radiotherapy	3.72 (0.56)	3.85 (0.48)	3.97 (0.37)	3.06 (0.75)	3.51 (0.39)	3.25 (0.75)
DGH	4.01 (0.52)	3.77 (0.48)	4.05 (0.46)	3.07 (0.74)	2.86 (0.56)	3.15 (0.73)
Pharmacy	3.34 (0.68)	3.59 (0.74)	3.88 (0.68)	3.16 (0.94)	2.96 (0.60)	3.36 (0.97)
Academic status						
Academic	3.65 (0.47)*	3.50 (0.50)	3.71 (0.47)*	2.91 (0.65)*	3.07 (0.49)*	3.37 (0.56)*
Non-academic	3.57 (0.63)*	3.45 (0.60)	3.61 (0.73)*	3.02 (0.76)*	2.87 (0.65)*	3.26 (0.67)*

*Note. N* = 1314; * Between group differences at *p* <0.05; *CCL* cardiac catheterization lab, *CCU - HFA* coronary care unit - heart first aid unit, *DGH* Department of Gastroenterology and Hepatology, *Perc. of Management* Perceptions of Management

**Fig. 1:**
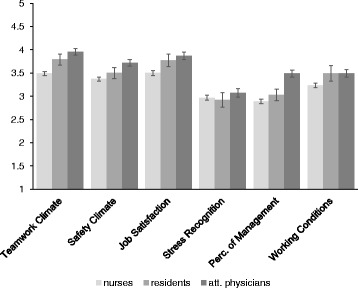
SAQ Means for Professional Position. Overview of mean scores and 95 % Confidence Intervals. *Att. physicians* attending physicians, *Perc. of Management* Perceptions of Management

Follow-up univariate ANOVA’s revealed that there was an effect of professional position on Teamwork Climate, *F*(3, 1155) = 49.08, *p* <0.001, η_p_^2^ = 0.11, on Safety Climate, *F*(3, 1155) = 22.63, *p* <0.001, η_p_^2^ = 0.06, on Job Satisfaction, *F*(3, 1155) = 23.69, *p* <0.001, η_p_^2^ = 0.06, on Perceptions of Management, *F*(3, 1155) = 2.95, *p* = .032, η_p_^2^ = 0.01, and on Working Conditions, *F*(3, 1155) = 13.63, *p* <0.001, η_p_^2^ = 0.03. An overview of means and confidence intervals is provided in Fig. 1.

A post-hoc Bonferroni test revealed that attending physicians were more positive about Teamwork Climate than both residents, *p* < 0.001, *d* = 0.35, and nurses, *p* <0.001, *d* = 0.90. Residents were more positive about Teamwork Climate than nurses, *p* <0.001, *d* = 0.59. For Safety Climate, attending physicians were more positive than residents, *p* = 0.008, *d* = 0.06, and nurses, *p* <0.001, *d* = 0.64. Furthermore, nurses experienced lower Job Satisfaction than attending physicians, *p* <0.001, *d* = 0.59, and residents, *p* = 0.001, *d* = 0.47. Finally, nurses were less positive about Working Conditions than attending physicians, *p* <0.001, *d* = 0.42, and residents, *p* = 0.001, *d* = 0.46.

**Fig. 2:**
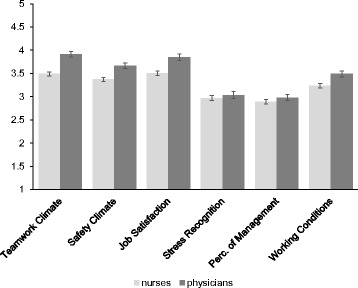
SAQ Means for Physicians versus Nurses. Overview of mean scores and 95 % Confidence Intervals. *Perc. of Management* Perceptions of Management

The follow-up univariate ANOVA’s concerning physicians vs. nurses revealed that physicians were more positive about Teamwork Climate than nurses, *F*(1, 945) = 111.90, *p* <0.001, η_p_^2^ = 0.12. Physicians were more positive about Safety Climate than nurses, *F*(1, 945) = 60.43, *p* <0.001, η_p_^2^ = 0.06. Physicians experienced more Job Satisfaction than nurses, *F*(1, 945) = 65.23, *p* <0.001, η_p_^2^ = 0.07. Physicians had higher Perceptions of Management than nurses, *F*(1, 945) = 4.73, *p* = 0.030, η_p_^2^ = 0.01. Finally, physicians were found to experience better Working Conditions than nurses, *F*(1, 945) = 30.12, *p* <0.001, η_p_^2^ = 0.04. An overview of means and confidence intervals is provided in Fig. 2.

**Fig. 3:**
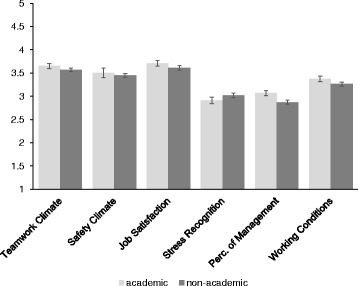
SAQ Means for Academic Status. Overview of mean scores and 95 % Confidence Intervals. *Perc. of Management* Perceptions of Management

Follow-up univariate ANOVA’s related to university status of the hospital revealed that university hospitals were more positive about Teamwork Climate than teaching hospitals, *F*(1, 1255) = 6.23, *p* = 0.013, η_p_^2^ = 0.01. Also, more Job Satisfaction was experienced in university hospitals than in teaching hospitals, *F*(1, 1255) = 7.28, *p* = 0.007, η_p_^2^ = 0.01. Scores on Stress Recognition were lower in academic hospitals than in teaching hospitals, *F*(1, 1255) = 6.91, *p* = 0.009, η_p_^2^ = 0.01. In university hospitals, Perceptions of Management were higher than in teaching hospitals, *F*(1, 1255) = 33.54, *p* <0.001, η_p_^2^ = 0.03. Finally, university health care providers from hospitals were more positive about Working Conditions than teaching hospitals, *F*(1, 1255) = 9.58, *p* = 0.002, η_p_^2^ = 0.01. An overview of means and confidence intervals is provided in Fig. 3.

### Reliability and correlation analysis

Reliability analysis of the SAQ-NL showed strong internal consistency, Cronbach’s α = .87, see Additional file 1. For the Perceptions of Management and Working Conditions categories Cronbach’s α’s were below the .70 reliability threshold (.65 and .57, respectively) though. Interestingly, in spite of having no effect on overall SAQ-NL reliability, exclusion of item 29 would result in the Working Conditions dimension reliability increasing from .57 to .70.

Teamwork Climate and Safety Climate were correlated at about .70. In addition, Stress Recognition was consistently negatively related to all other categories (see Table 3). The complete dataset is available as Additional file 2.

**Table 3:** SAQ-NL Dimension Means and (Standard Deviations), Correlations, and Cronbach’s α’s

	Mean (*SD*)	1.	2.	3.	4.	5.	6.
1. Teamwork Climate	3.60 (0.58)	**.76**					
2. Safety Climate	3.47 (0.57)	.73	**.77**				
3. Job Satisfaction	3.65 (0.65)	.56	.54	**.84**			
4. Stress Recognition	2.99 (0.73)	-.14	-.15	-.15	**.69**		
5. Perc. of Management	3.01 (0.66)	.33	.36	.40	-.17	**.65**	
6. Working Conditions	3.13 (0.56)	.47	.48	.40	-.18	.35	**.57**

*Note. N* = 1314; *Perc. of Management* perceptions of management, All correlations are significant at the *p* <0.01 level; Cronbach’s α’s appear in boldface on the diagonal

## Discussion

We developed and refined a Dutch language version of the SAQ and used it on a broad sample of hospital departments in the Netherlands. CFA confirmed the appropriateness of the proposed model and the resulting psychometric properties were good for this instrument. Internal consistency as well as correlations were similar to the results published by Sexton and colleagues (2006) in their validation study of the SAQ [10].

Furthermore, reference data were reported for comparison purposes. In a pattern of results quite similar to what has been found in other translations of the SAQ [15, 23], the SAQ-NL was associated with significant unit-level variability, higher scores for physicians than non-physicians, and psychometrically valid scales.

Explorative analyses of the data revealed two interesting findings. First, the robust finding that physicians score higher in five out of six SAQ-NL domains than nurses is consistent with previous research [24]. This represents a different perception of the safety climate within clinical teams, a factor that should be taken into account during human factors awareness training. Second, university hospitals were found to be slightly more positive about safety climate than non-university teaching hospitals. A possible explanation might be the lower clinical production pressure perceived in the academic setting, as well as a teaching environment with more emphasis on supervision. However, university hospitals scored slightly lower in stress recognition. We can offer no explanation for this finding. Several studies find that the SAQ-factor Stress Recognition has problems regarding construct validity and that it does not vary significantly between organizational units [25].

### Strengths

The first strength of the present study is the broad spectrum of participating hospitals, departments and professionals resulting in a sample that could be considered a representative cross section of acute and critical care departments in the Dutch clinical healthcare setting. In addition, the large sample size resulted in sufficient representation of professionals in the categories utilized in this study. Thirdly, as this study provides an open source Dutch translation of the SAQ short form, it may serve as a basis for future research. This would allow for better comparison of future investigations into safety climate in hospital departments in the Netherlands.

### Limitations

The most important limitation of the present study is the fact that hospital departments were not a random sample. The SAQ-NL was determined in units that were to receive human factors training, and it is therefore possible that these non-random units had safety culture norms that were not representative. One could argue that the fact that they signed up for human factors training could be the result of priority given to safety climate resulting in a higher safety culture norm than expected, or the opposite, that these departments wished to participate because of perceived problems with safety. A brief comparison of our overall means to other samples suggests that the latter was not the case. Nevertheless this would not impact the psychometric results, which ranged from adequate to good.

Second, in spite of our efforts to include as many different departments and clinical specialties as possible, we recognize this study cannot encompass the total clinical spectrum. We therefore encourage further research covering even more clinical specialties inside and outside of inpatient settings.

Third, item 16 (“this is a good place to work”) did not appear in the questionnaire initially and therefore resulted in a MV of 50 %. However, the large sample size limits the impact of this omission.

Finally, this study period covered 5 years. Possible effects of general changes in perceptions of clinical safety climate during this timeframe cannot be excluded. Nevertheless, results from the first 2 years compared to the last 2 years did not yield significant differences (data not shown), indicating that this is not likely to be an issue.

Perceived safety climate is associated with safety outcomes in hospital settings [26].

Therefore, determination of safety climate is of clinical relevance. The SAQ-NL in its present form shows promise to be a benchmarked tool for future research into patient safety. Exclusion of item 29 “All the necessary information for diagnostic and therapeutic decisions is routinely available to me” would result in an increase of Working Conditions dimension reliability (from .57 to .70). Even though this would not impact overall SAQ-NL reliability, adapting, deleting, or at the very least, monitoring this item is something to consider in future research that utilizes the SAQ-NL. After this adjustment psychometric properties should be reassessed in a randomly selected sample and hospitals and departments prior to more widespread use in Dutch hospital settings.

## Conclusions

We assessed the psychometric properties of the Dutch language version of the SAQ, the SAQ-NL, and provided insight into safety climate in a variety of clinical departments in Dutch hospitals. The SAQ-NL is a reliable instrument to measure safety climate in the Dutch hospital setting. Further research is needed to validate the SAQ-NL as a monitoring tool for pre-and-post administration of the impact of interventions related to safety climate.

## Additional files

Additional file 1: SAQ Items, Translations, Response Rates, Means, Standard Deviations, Factor Loading, and Reliability Characteristics. (DOCX 76 kb) Additional file 2: Research Dataset. (XLSX 687 kb)

## Abbreviations

AMOS: Analysis of MOment Structures
ANOVA: Univariate analysis of variance
CCL: Cardiac Catheterization Lab
CCU-HFA: Coronary Care Unit - Heart First Aid unit
CFA: Confirmatory Factor Analysis
CRM: Crew Resource Management
DGH: Department of Gastroenterology and Hepatology
ER: Emergency Room
ICU: Intensive Care Unit
MCU: Medium Care Unit
OR: Operating Room
RTX: Radiotherapy
SAQ: Safety Attitudes Questionnaire
SAQ-NL: Dutch language version of the SAQ
