# Supplementary material for: Validation of the Dutch language version of the Safety Attitudes Questionnaire (SAQ-NL)
Source: BMC Health Serv Res. 2016 Aug 15;16:385. doi: 10.1186/s12913-016-1648-3 (PMC4986249; doi:10.1186/s12913-016-1648-3)
Supplement: Additional file 1: — SAQ Items, Translations, Response Rates, Means, Standard Deviations, Factor Loading, and Reliability Characteristics. (DOCX 76 kb) [file 12913_2016_1648_MOESM1_ESM.docx]

**Additional file 1**

*SAQ Items, Translations, Response Rates, Means, Standard Deviations, Factor Loading, and Reliability Characteristics*

| Dimensions and questions | Missing  (%) | Mean (*SD*) | Disagree/agree  (%) | Corrected item-total correlation | CFA factor loadings | α if item deleted (dimension) |
| --- | --- | --- | --- | --- | --- | --- |
| **Total scale α = .87** |  |  |  |  |  |  |
| **Teamwork Climate** |  |  |  |  |  | **.76** |
| 1. Nurse input is well received in this clinical area.  *De inbreng van verpleegREkundigen wordt op mijn unit op prijs gesteld.* | 3.7 | 3.76 (0.84) | 7.0 / 65.8 | .49 | .78 | .73 |
| 2. In this clinical area, it is difficult to speak up if I perceive a problem with patient care.  *Op mijn unit is het moeilijk om het uit te spreken als ik merk dat er een probleem is met de patiëntenzorg.* | 3.5 | 2.53 (0.93) | 14.8 / 52.4 | .40 | .78 | .75 |
| 3. Disagreements in this clinical area are resolved appropriately (i.e., not *who* is right, but *what* is best for the patient).  *Meningsverschillen op mijn afdeling worden op een goede manier opgelost (d.w.z. niet wie heeft er gelijk, maar wat is het beste voor de patiënt).* | 3.4 | 3.26 (0.87) | 16.6 / 40.1 | .65 | 1.12 | .71 |
| 4. I have the support I need from other personnel to care for patients.  *Ik krijg de ondersteuning die ik nodig heb van staf-artsen om voor patiënten te kunnen zorgen.* | 3.7 | 3.65 (0.84) | 8.2 / 59.5 | .57 | .88 | .71 |
| 5. It is easy for personnel here to ask questions when there is something that they do not understand.  *Medewerkers op mijn unit kunnen gemakkelijk vragen stellen als er iets is dat ze niet begrijpen.* | 3.3 | 3.96 (0.76) | 3.8 / 76.6 | .57 | .86 | .72 |
| 6. The physicians and nurses here work together as a well-coordinated team.  *De artsen en de rest van het team hebben hier een goede samenwerking.* | 4.1 | 3.48 (0.89) | 11.9 / 48.1 | .53 | 1.00 | .72 |
| **Safety Climate** |  |  |  |  |  | **.77** |
| 7. I would feel safe being treated here as a patient.  *Als ik hier als patiënt zou worden behandeld, zou ik me veilig voelen.* | 3.6 | 3.78 (0.79) | 5.4 / 67.2 | .56 | .80 | .73 |
| 8. Medical errors are handled appropriately in this clinical area.  *Medische fouten worden goed afgehandeld op de afdeling.* | 5.0 | 3.38 (0.84) | 11.3 / 43.0 | .56 | .90 | .73 |
| 9. I know the proper channels to direct questions regarding patient safety in this clinical area.  *Ik weet aan wie ik vragen kan stellen als het gaat om de patiëntveiligheid op de afdeling waar ik werk.* | 4.0 | 3.63 (0.94) | 11.6 / 60.8 | .37 | .62 | .76 |
| 10. I receive appropriate feedback about my performance.  *Ik krijg goede feedback op mijn functioneren.* | 3.6 | 3.29 (0.92) | 18.0 / 42.5 | .45 | .74 | .74 |
| 11. In this clinical area, it is difficult to discuss errors.  *Op de unit waar ik werk is het lastig om fouten te bespreken.* | 3.0 | 2.53 (0.93) | 15.4 / 52.7 | .52 | .92 | .73 |
| 12. I am encouraged by my colleagues to report any patient safety concerns I may have.  *Ik word door mijn collega’s aangemoedigd al mijn bedenkingen wat patiëntveiligheid betreft te melden.* | 3.4 | 3.43 (0.86) | 12.3 / 46.8 | .45 | .70 | .74 |
| 13. The culture in this clinical area makes it easy to learn from the errors of others.  *De cultuur op mijn unit maakt het makkelijk om van fouten van anderen te leren.* | 3.3 | 3.31 (0.86) | 15.4 / 42.2 | .59 | 1.00 | .71 |
| **Job Satisfaction** |  |  |  |  |  | **.84** |
| 14. I like my job.  *Ik ben enthousiast over mijn baan.* | 5.3 | 4.09 (0.78) | 3.3 / 78.3 | .56 | .95 | .80 |
| 15. Working here is like being part of a large family.  *Het werken in dit ziekenhuis voelt als deel uit maken van een grote familie.* | 6.5 | 2.99 (0.91) | 22.7 / 25.7 | .48 | 1.01 | .82 |
| 16. This is a good place to work.  *Dit ziekenhuis is een goede plek om te werken.* | 50.0 | 3.80 (0.91) | 4.6 / 35.1 | .57 | .99 | .80 |
| 17. I am proud to work in this clinical area.  *Ik ben trots dit ik in dit ziekenhuis werk.* | 5.6 | 3.87 (0.88) | 6.6 / 69.4 | .63 | 1.20 | .77 |
| 18. Morale in this clinical area is high.  *Het moreel op deze afdeling is hoog.* | 4.8 | 3.53 (0.98) | 14.2 / 55.3 | .58 | 1.00 | .83 |
| **Stress Recognition** |  |  |  |  |  | **.69** |
| 19. When my workload becomes excessive, my performance is impaired.  *Wanneer mijn werkdruk te hoog wordt, dan lijdt mijn functioneren daaronder.* | 4.0 | 3.23 (1.05) | 26.3 / 41.7 | -.15 | 1.16 | .62 |
| 20. I am less effective at work when fatigued.  *Als ik vermoeid ben dan verricht ik routinetaken minder goed.* | 6.8 | 2.89 (0.96) | 34.6 / 26.1 | -.06 | .99 | .64 |
| 21. I am more likely to make errors in tense or hostile situations.  *Ik ben meer geneigd om fouten te maken in een gespannen of bedreigende situatie.* | 3.8 | 2.99 (1.00) | 32.2 / 30.4 | -.04 | 1.10 | .63 |
| 22. Fatigue impairs my performance during emergency situations.  *Vermoeidheid hindert mijn functioneren tijdens acute situaties.* | 4.9 | 2.82 (0.96) | 36.6 / 24.1 | -.09 | 1.00 | .63 |
| **Perceptions of Management** |  |  |  |  |  | **.65** |
| 23. Management supports my daily efforts.  *Het ziekenhuismanagement helpt me bij mijn dagelijkse bezigheden.* | 5.3 | 2.70 (0.87) | 34.0 / 13.9 | .42 | 2.04 | .56 |
| 24. Management doesn’t knowingly compromise patient safety.  *Het ziekenhuismanagement brengt de veiligheid van de patiënten niet bewust in gevaar.* | 5.4 | 3.54 (0.88) | 10.0 / 52.5 | .39 | 1.59 | .62 |
| 25. Problem personnel are dealt with constructively by our unit / hospital management.  *Dit ziekenhuis gaat constructief om met minder goed functionerend personeel.* | 5.8 | 2.63 (0.82) | 35.8 / 9.2 | .44 | 1.59 | .61 |
| 26. I get adequate, timely info about events that might affect my work, from unit / hospital management.  *Ik krijg voldoende, tijdige informatie over gebeurtenissen in het ziekenhuis die invloed kunnen hebben op mijn werk.* | 4.7 | 2.93 (0.94) | 29.3 / 27.5 | .53 | 2.53 | .53 |
| 27. The levels of staffing in this clinical area are sufficient to handle the number of patients.  *We hebben genoeg personeel om de werklast aan te kunnen.* | 5.2 | 2.89 (1.08) | 34.5 / 31.4 | .24 | 1.00 | .67 |
| **Working Conditions** |  |  |  |  |  | **.57** |
| 28. This hospital does a good job of training new personnel.  *Dit ziekenhuis is goed in het trainen van nieuw personeel.* | 5.3 | 3.20 (0.82) | 14.5 / 32.6 | .47 | .93 | .33 |
| 29. All the necessary information for diagnostic and therapeutic decisions is routinely available to me.  *Ik beschik steeds over alle informatie die nodig is voor diagnostische en therapeutische beslissingen.* | 5.5 | 3.18 (0.85) | 18.7 / 34.3 | .36 | .36 | .70 |
| 30. Trainees in my discipline are adequately supervised.  *Degenen die opgeleid worden in mijn discipline krijgen voldoende begeleiding.* | 4.6 | 3.50 (0.90) | 13.5 / 55.1 | .43 | 1.00 | .31 |

*Note*. *N* = 1314; Total scale and dimension Cronbach’s α’s appear in boldface; Dutch translated items appear in italics underneath original item. Items 2 and 11 are reverse scored.
